# A statistical framework for revealing signaling pathways perturbed by DNA variants

**DOI:** 10.1093/nar/gkv203

**Published:** 2015-03-12

**Authors:** Roni Wilentzik, Irit Gat-Viks

**Affiliations:** Department of Cell Research and Immunology, The George S. Wise Faculty of Life Sciences, Tel Aviv University, 6997801 Tel Aviv, Israel

## Abstract

Much of the inter-individual variation in gene expression is triggered via perturbations of signaling networks by DNA variants. We present a novel probabilistic approach for identifying the particular pathways by which DNA variants perturb the signaling network. Our procedure, called PINE, relies on a systematic integration of established biological knowledge of signaling networks with data on transcriptional responses to various experimental conditions. Unlike previous approaches, PINE provides statistical aspects that are critical for prioritizing hypotheses for followup experiments. Using simulated data, we show that higher accuracy is attained with PINE than with existing methods. We used PINE to analyze transcriptional responses of immune dendritic cells to several pathogenic stimulations. PINE identified statistically significant genetic perturbations in the pathogen-sensing signaling network, suggesting previously uncharacterized regulatory mechanisms for functional DNA variants.

## INTRODUCTION

With the advent of transcription profiling technologies, it is now possible to identify genetic associations between DNA variants and gene-expression traits (1,2). Such methodology, called expression Quantitative Trait Loci (eQTL) analysis, is highly successful in identifying DNA variants and their ‘associated genes’ (3), but cannot as yet reveal the mechanisms translating between a variant and transcriptional diversity in a population. A critical prerequisite for understanding expression diversity is knowledge of the signaling pathways through which DNA variants perturb the functionality of the molecular network. In this article we refer to a signaling pathway as a ‘branch’ within a network; a ‘perturbed branch’ is a network branch whose functionality is altered as a consequence of direct interaction with a DNA variant.

Several approaches have been developed to identify the perturbed branch of a DNA variant within a given interaction network. The underlying assumption in these methods is that the network of protein–protein and protein–DNA interactions spread the signals from the perturbed branch toward the associated genes. Accordingly, for each variant these algorithms prioritize network branches that are proximal to the associated genes (e.g. using a random walk model (4) or an electric circuit (5)). Although these methods perform well when applied on eQTL data measured in a single condition (e.g. a baseline cell state), their biological relevance is limited in the case of changing environments. In particular, when these algorithms are used to identify perturbed branches, the assumption is that the associations hold under all experimental stimulations, whereas in fact, genes are found to associate only in a subset of these stimulations (6).

In attempting to identify the perturbed branch of a DNA variant in a given network, both the position of the associated genes in the network and the stimulus specificity of the associations should be considered. We recently developed a powerful approach called InCircuit, which utilizes eQTL data across multiple stimulations to improve the quality of predictions (6). InCircuit relies on a typical signaling network that transfers environmental stimulations through a series of reactions and interactions. Using the known positions of the stimulation components within this network, it is possible to infer the set of stimulations whose signals are transferred through each of the network's branches. Given a variant, InCircuit predicts one or more perturbed branches based on a full agreement between (i) the subset of stimulations in which the target genes associate with the variant and (ii) the subset of stimulations whose signals propagate through the network branch. This deterministic approach provides a list of predicted perturbed branches but cannot assess the statistical significance of these predictions.

**Figure 1.**
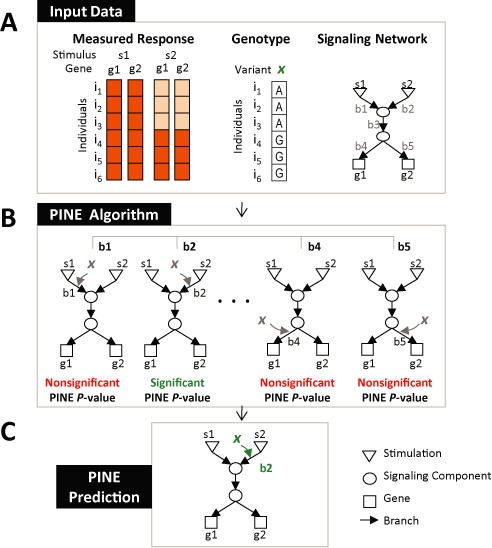
An overview of the PINE approach. (**A**) PINE takes as input transcriptional responses of multiple genes (*g_1_*, *g_2_*) under several stimulations (*s_1_*, *s_2_*) across a population of individuals (*i_1_*−*i_6_*; left). Each individual is further accompanied by genotyping data of the DNA variant under study (variant *x*; middle; since we focus on fully homozygous lines, the allele in one chromosome is sufficient). In addition, PINE takes as input a signaling network (right). (**B**) For each candidate perturbed branch (*b_1_*−*b_5_*) PINE calculates a statistical significance score, the ‘PINE *P*-value’ score, which evaluates the match between model predictions, genotyping data and transcriptional response measurements. PINE *P*-value calculations of candidate perturbed branches *b_2_* and *b_4_* are detailed in Figure 2. (**C**) In the exemplified case, only a single high-scoring branch exists. The output is therefore an extended signaling network model that includes an embedding of variant *x* within the network branch *b_2._* When multiple high-scoring branches exist, the PINE framework allows additional analyses (not shown in this illustration) to resolve the best-fit perturbed branch.

Here we present PINE (‘Perturbations In NEtworks’), an algorithm that combines prior knowledge about a signaling network together with transcription data across several stimulations and multiple genotyped individuals so as to provide statistically significant hypotheses about network branches perturbed by particular DNA variants (Figure 1). Our algorithm assumes that DNA variants affect the functionality of network branches when transmitting stimulation signals and that the network positions of all transcribed genes associating with these variants are known. PINE is currently designed for the analysis of fully homozygous recombinant inbred strains that are commonly investigated in genetic studies (1). Notably, several benchmarks indicate the high quality of the PINE algorithm. First, we demonstrate the good performance of the PINE method on simulated data, outperforming existing methods. Secondly, we demonstrate PINE's robustness in the case of erroneous prior knowledge about the transcribed genes in the signaling network. Finally, we applied PINE to gene-expression data in a large population of (genotyped) recombinant inbred BXD mouse strains (7,8), obtained during the *in vitro* response of immune bone marrow-derived dendritic cells (DCs) to three pathogenic-like stimulations: synthetic triacylated lipoprotein Pam3CSK4 (PAM), lipopolysaccharide (LPS) and polyinosinic-polycytidylic acid (poly I:C) (6). These stimulations affect the Toll-like and Retinoic acid-like receptors (TLR/RLR), and share downstream network branches and transcribed genes along several inflammatory and antiviral signaling pathways (9,10). Using this network as input, PINE suggests a single most-significant perturbed branch for four different variants, thereby providing an in-depth view of inherited variation in the mammalian TLR/RLR signaling network.

## MATERIALS AND METHODS

### Overview of the PINE algorithm

We developed PINE, a probabilistic graphical model (11) to reconstruct the network branch perturbed by a particular DNA variant. Our model is focused on DNA variants that alter the propagation of responses to stimulations, but have no effect in the absence of stimulation. Given a certain variant, the input for the PINE algorithm should include: genotyping data for the DNA variant across a group of individuals from a given collection of fully homozygous lines (Figure 1A, middle); a list of environmental stimulations; a group of genes that all associate with the requested variant following at least one of the environmental stimulations under study; and transcription responses of all genes in this group, following each of the input stimulations and across the same cohort of individuals (Figure 1A, left). In particular, such transcriptional ‘responses’ are defined as the changes (increase or decrease) in the levels of gene expression following stimulation, relatively to the baseline gene expression levels.

Another important input for the PINE algorithm is a detailed predictive model of a signaling network that mediates transcriptional responses to stimulations (Figure 1A, right). The network's components (nodes) are the stimulations, the associated genes and the proteins that mediate between them; branches (edges) in this network represent the influence of one component on another component in response to stimulations. Similarly to standard predictive modeling approaches, the network describes how each of the stimuli triggers a certain response signal that propagates through the network until it influences the transcriptional responses of its downstream genes. When adding a variant to a certain branch, the network can also describe how the alleles of this variant determine (‘perturb’) the propagation of signals through this branch, which in turn trigger inherited variation in the transcriptional responses of downstream genes (see demonstration in Supplementary Figure S1).

Given these inputs, the PINE algorithm searches exhaustively for a perturbed branch that can explain the measured transcriptional responses to various stimulations across individuals and across genes (Figure 1B and C). The underlying rationale of PINE is that when the causal DNA variant is added to affect a particular branch, it improves the match between the model's predicted responses and the measured responses of the differently genotyped individuals. Based on this premise, the ‘PINE *P*-value’ evaluates the fit between model predictions and measurements and is used to guide the search for significant hypotheses. All branches that attain PINE *P*-values that are lower than a certain threshold are referred to as *‘*significantly-perturbed branches’.

**Figure 2.**
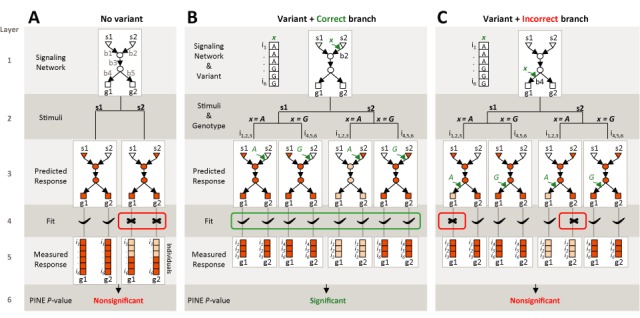
An illustration of the PINE *P*-value score (assuming the input data from Figure 1 with a single correct branch *b_2_*). The input of the calculation includes a known network (first layer) along with the measured responses of multiple genes, individuals and stimulations (fifth layer). The third layer shows the predicted responses of the network-regulated genes under each of the variant's genotypes and the stimulations triggering the network, as depicted in the second layer. High/low predicted and measured responses are in dark/light orange. The PINE *P*-value (sixth layer) is calculated on the basis of the goodness of fit between the predicted and measured responses (fourth layer). (**A**) A general network model without any DNA variant. Following stimulation *s­_2_*, individuals *i_1_−i_3_* have low responses compared to the high responses of individuals *i_4_−i_6_* (fifth layer), producing a non-significant PINE *P*-value. (**B**,**C**) A network model harboring a variant perturbing the correct branch *b_2_* (B) or an incorrect branch *b_4_* (C). When a branch is perturbed by a DNA variant, the model's predictions consider not only the different stimuli but also the distinct genotypes. Using branch *b_2_*, but not branch *b_4_*, a good fit can be attained and the PINE *P*-value score is improved, allowing correct identification of the perturbed *b_2_* branch.

As an example, Figure 2 provides a detailed illustration showing how the PINE *P*-value score is attained in the dataset presented in Figure 1A. In this dataset, each individual carrying the *‘AA’* genotype in a variant *x* shows a lower response of genes *g_1_* and *g_2_* to stimulation *s_2_* compared to those individuals carrying the *‘GG’* genotype. Such an effect is observed under stimulation *s_2_* but not under stimulation *s_1_* (Figure 1A, left). Given a stimulus, it is possible to determine the predicted response of each network component and examine the fit of these predictions to the measured transcriptional responses of the genes. Notably, the general model, which does not include the functional variant *x*, cannot successfully predict the entire population and the result is a poor PINE *P*-value (in this case, high predicted responses in all genes and stimuli, which do not fit the low responses of individuals *i*_*1*_*−i*_*3*_ to the *s_2_* stimulus; Figure 2A). However, a model that includes variant *x* interacting with branch *b_2_* fits well with the response data and yields a highly significant PINE *P*-value (Figure 2B). Upon interaction of variant *x* with a wrong branch, which has a different profile of triggering stimuli and downstream genes, the resulting *P*-value is non-significant because of the lack of fit between model predictions and data (e.g. branch *b*_*4*_; Figure 2C). Taken together, the PINE algorithm generates hypotheses of the form ‘DNA variant *x* perturbs a certain component within a network branch *b_2_*, which is triggered by stimulation *s_2_* and affects its downstream signaling branches *b_3_, b_4_, b_5_* and the transcriptional responses of downstream genes *g_1_* and *g_2_*’ (Figures 1C and 2B).

The analysis of network branches falls in one of three main classes. In the first class, a network includes only a single branch that improves the fit between model predictions and data (e.g. Figures 1 and 2), suggesting a single mechanism for the network perturbation. The second class considers the presence of two or more highly-significant perturbed branches, where none of these branches outperforms the remaining branches. This may reflect several branches that cannot be prioritized, possibly due to multiple variant-network interactions, or alternatively, an incomplete prior network model or multiple branches that act in a degenerate fashion. The third class also consists of several significantly-perturbed branches, but in this case, one of the branches outperforms the remaining branches; this suggests that though several branches propagate the variation in response, only one *‘*leading branch’ directly interacts with the variant and hence likely provides a better model compared to all other branches. As the leading branch may not be apparent using the PINE *P*-value score (which cannot be used to compare different branches), we further suggest a ‘leading *P*-value’ score that is capable of directly comparing two candidate branches.

Both the PINE and the leading scores are required to achieve a comprehensive view of perturbed branches: whereas the second class of branches can only be captured by the PINE *P*-value score, the leading *P*-value score is essential for identifying the leading branches of the third class. In accordance, the PINE framework is applied as follows: we first calculate the PINE *P*-values (as exemplified in Figure 1A and B) thus identifying significantly perturbed branches that fall in all three classes. In case of multiple branches with highly significant PINE *P*-values, further prioritization can be achieved using the leading *P*-value score.

### The PINE algorithm

#### The PINE input

Given a certain variant *x* and a collection of *m* individuals $I = \{ i_{1,} ,...,i_m \}$, PINE takes as input three types of data:

- Transcriptional response data for a given group of genes $G(x) = \{ g_{1,} ,...,g_t \}$, such that *t* is the number of genes that are associated with variant *x*. We indicate the response dataset for the gene group *G*(*x*) by *R^G^*^(^*^x^*^)^. This dataset includes the responses of all genes in *G*(*x*) following a group of *n* stimulations measured across all *m* individuals. The ‘response’ of a gene following a stimuli in a given individual is defined as the log ratio between that gene's expression level after stimulation and its baseline expression level. Importantly, each measurement in *R^G^*^(*x*)^ is annotated with the actual experiment that was performed, encompassing the combination of states of all *n* stimulation variables, referred to as a ‘stimulation configuration’ $S = \{ s_{1,} ,...,s_n \}$. A stimulation state can be either 0 (absence of stimulation) or 1 (presence of stimulation). For example, if our dataset consists of three different stimulations, the configurations would be {1,0,0}, {0,1,0} and {0,0,1}.
- Genotyping data of the causal DNA variant *x* of group *G*(*x*) across the same *m* individuals, where *x*(*i*) denotes the genotype of variant *x* in individual *i*. Here $x(i) \in \{ a,\bar a\}$assuming fully homozygous lines (i.e. two possible genotypes for each variant).
- Signaling network model *M*^G^^(^^x^^)^ consisting of the stimulation variables, signaling variables, the group of genes *G*(*x*) and the regulatory relations between the variables. The model is based on prior knowledge about the biological components and the relationships among them. To formalize the network we utilized a Bayesian network modeling approach that extends the MetaReg approach (12,13). The input signaling network is constructed in two steps. In step 1 we define variables and states. In particular, the network consists of three types of components: the environmental ‘stimulations’, which trigger ‘signaling components’ (such as receptors and transcription factors) that subsequently control changes in the transcriptional responses of downstream ‘genes’ (e.g. Figure 1A, right). A fourth type of component, the ‘DNA variant’, is then added to the model as part of the PINE algorithm (e.g. Figure 1B). Each component may attain one of several discrete ‘states’: a DNA variant can be in one of two states, corresponding to its two genotypes ($a$ or $\bar a$). A stimulation variable also has two states, indicating whether the environmental stimulation is applied or not. A signaling component or a gene can be in one of several ‘response states’, representing the activity (of the signaling component) or the transcriptional response (of the gene). In this study we focus on stimulation-specific variants, which have no effect in the absence of an upstream stimulation and may only perturb the propagation of response to stimulation signal through a signaling branch. In accordance, all signaling components and genes can be in one of three response states, referred to as the ‘no-response’, ‘$a$-response’ and ‘$\bar a$-response’ states: the no-response state refers to the absence of stimulation, whereas the $a$- and $\bar a$-response states refer to a response to stimulation that is determined by the particular genotype of the perturbing variant.

In step 2, for each variable we define a ‘regulation function’ that describes how the state of a given variable changes with the changing state of its regulatory variables. In particular, each variable is associated with a certain set of regulatory variables and a deterministic regulation function (e.g. Supplementary Figure S2, left and middle). The deterministic function is then translated into a conditional probability function, which represents our confidence as to the biological assumptions through the ‘network confidence level’ *β* parameter (Supplementary Figure S2, right). Notably, in our simplified graphical representations (e.g. Figures 1 and 2) the nodes correspond to variables and the branches to regulatory relationships among the variables, but the underlying regulatory functions are omitted.

#### Formalizing the effect of a DNA variant on the functionality of a branch

To test the effect of a DNA variant on the functionality of each branch, we further extend the input network model. For each branch we define a ‘proxy node’, which is a predecessor or successor variable of the branch, such that the out- or in-degree (respectively) of the proxy is exactly 1. If no proxy node exists (i.e. both the out-degree of the direct predecessor and the in-degree of the direct successor are >1), an intermediate mock node will be introduced within that branch and will serve as a proxy. The effect of a DNA variant on the functionality of a branch is modeled by altering the regulatory function of its proxy node. Several types of such alterations are considered. First, the variant may have ‘no effect’ on the proxy node, i.e. individuals carrying distinct genotypes will attain the same state. In contrast, a certain genotype may increase or decrease the activity of the proxy node, meaning that one genotype will lead to a higher or lower activity level than the other genotype. We refer to such alterations as the ‘perturbation effect’ (in short, ‘effect’): effects of ‘type $a$’ and ‘type $\bar a$’ refer to a higher activity in $a$- or in $\bar a$-carrying individuals, respectively. Collectively, we define the three types of effects as *E* = {no effect, type $a$, type $\bar a$}.

#### Formalizing the likelihood function

Let $M_{x,b,e}^{G(x)}$ be the network *M^G^*^(^*^x^*^)^ after adding the DNA variant *x* to perturb a branch $b \in B$, assuming perturbation effect $e \in E$ (where *B* is the collection of branches and *E* is the group of effect types). The likelihood of data *R^G^*^(*x*^^)^ and variant *x* given the model $M_{x,b,e}^{G(x)}$ is defined as:

(1) \begin{equation*} L(R^{G(x)} |M_{x,b,e}^{G(x)} ,\theta ^{G(x)} ) = \prod _{g \in G(x)} \prod _{s \in S} \prod _{i \in I} L(R_i^{g,s} |M_{x,b,e}^{G(x)} ,\theta ^g ) \end{equation*}

where $R_i^{g,s}$ is the ‘measured response’ of gene *g* following stimulation configuration *s* in individual *i*. We assume that each gene *g* is modeled by a mixture of three Gaussians that correspond to its three transcriptional response states: the no-response,$a$- and $\bar a$-response Gaussians refer to the no-response state (in the absence of stimulation) as well as $a$- and $\bar a$-response states in $a$- and $\bar a$-carrying individuals, respectively; these three response states and three Gaussians are hereby denoted $K = \{ 0,a,\bar a\}$, where $\theta ^{G(x)} = \{ \theta ^g |g = 1,...,t\}$ and $\theta ^g = \{ \mu _0^g ,\mu _a^g ,\mu _{\bar a}^g ,\sigma _0^g ,\sigma _a^g ,\sigma _{\bar a}^g \}$ are the means and variances of the three Gaussians associated with a gene *g*. The likelihood of each measured response is:

(2) \begin{equation*} L(R_i^{g,s} |M_{x,b,e}^{G(x)} ,\theta ^g ) = \sum _{k \in K} P_{x(i)}^{g,s} (k) \cdot f(R_i^{g,s} ,\mu _k^g ,\sigma _k^g ) \end{equation*}

where *f* is the normal probability density function and the mixture coefficient $P_{x(i)}^{g,s} (k)$ is the ‘predicted response’. A predicted response $P_{x(i)}^{g,s} (k)$ denotes the probability that variable gene *g* will have a response state $k \in K$ following the stimulation configuration *s* in individual *i* carrying genotype *x*(*i*) in the DNA variant *x*. The stimulation configuration and the genotypic state of the variant are the observed variables used to infer the predicted responses for each genotype and gene level, based on an inference algorithm applied to $M_{x,b,e}^{G(x)}$ (here we used a variable elimination procedure (11) using the ‘Bayes Net Toolbox’ (BNT) implemented for Matlab (14)). Note that the model's predicted responses $P_{x(i)}^{g,s} (k)$ do not use information about the measured response. Since the predicted responses are independent of the data, the likelihood function allows a reliable comparison between model predictions and measured responses.

The predicted responses are used to calculate $\hat \theta _k^g = \{ \hat \mu _k^g ,\hat \sigma _k^g \}$, the maximum likelihood estimators for response state *k* in gene *g* (*g* = *1,..,t* and *k = 0, a*, $\bar a$):

(3) \begin{equation*} \begin{array}{*{20}l} {\hat \mu _k^g = \frac{{\sum _{s \in S} \sum _{i \in I} R_i^{g,s} \cdot P_{x(i)}^{g,s} (k)}}{{\sum _{s \in S} \sum _{i \in I} P_{x(i)}^{g,s} (k)}},} \\ {\hat \sigma _k^g = \sqrt {\frac{{\sum _{s \in S} \sum _{i \in I} (R_i^{g,s} - \hat \mu _k^g )^2 \cdot P_{x(i)}^{g,s} (k)}}{{\sum _{s \in S} \sum _{i \in I} P_{x(i)}^{g,s} (k)}}} } \\ \end{array} \end{equation*}

#### The PINE procedure

We next define a generalized likelihood ratio (LR) test that compares two hypotheses with respect to the existence (or non-existence) of a perturbation effect of a candidate variant *x* in a branch *b*. The null hypothesis assumes that the DNA variant does not affect the branch (in other words, the ‘no effect’ perturbation applies). The alternative hypothesis assumes that the DNA variant does affect the branch, through the perturbation effect of either *‘*type *a*’ or ‘type $\bar a$’. A significant LR value is obtained only if the effect of the variant on the branch improves the fit of the model to the measured data. Formally,

(4) \begin{equation*} \begin{array}{*{20}l} {LR(b |x, R^{G(x)} , M_{x,b}^{G(x)} ) = } \\ {\frac{{\max _{\theta ^{G(x)} , e \in E } L(R^{G(x)} |M_{x,b,e}^{G(x)} ,\theta ^{G(x)} )}}{{\max _{\theta ^{G(x)} ,e = no-effect } L(R^{G(x)} |M_{x,b,e}^{G(x)} ,\theta ^{G(x)} )}}} \\ \end{array} \end{equation*}

where for each effect type $e \in E$ we consider a different model $M_{x,b,e}^{G(x)}$ that is used to optimize *θ^G^*^(^*^x^*^)^ as detailed in Equation no. 3. The statistical significance of this LR value is calculated empirically by repeatedly permuting the labels of strains and re-calculating the LR values for the permuted dataset. The *P*-value is the fraction of permutation-based LR values that are at least as extreme as the original LR values. These *P*-values are referred to as the ‘PINE *P*-value*s*’. A significant PINE *P*-value for a particular branch *b* and a variant *x* suggests an effect of the variant on a certain component along this branch; such a branch is termed a ‘significantly-perturbed branch’.

In some cases two or more branches may attain significant PINE *P*-values for the same variant. A best-fit branch can then be revealed using an additional statistical test that compares two alternative branches *b’* and *b’’* for the same variant *x*:

(5) \begin{equation*} \begin{array}{*{20}l} {LR(b',b'' |x, R^{G(x)} , M_{x, b'}^{G(x)} , M_{x, b''}^{G(x)} ) = } \\ {\frac{{\max _{\theta ^{G(x)} , e \in E } L(R^{G(x)} |M_{x, b',e}^{G(x)} ,\theta ^{G(x)} )}}{{\max _{\theta ^{G(x)} , e \in E } L(R^{G(x)} |M_{x, b'',e}^{G(x)} ,\theta ^{G(x)} )}}} \\ \end{array} \end{equation*}

We use this LR statistic to calculate a ‘leading *P*-value’ based on a permutation test (reshuffling the labels of strains). A branch is called a ‘leading branch’ if its least significant leading *P*-value (when compared to all remaining branches) is significant. Notably, the leading *P*-value is designed for the case of a single branch outperforming the remaining branches; its utility is therefore limited for the case of several degenerated branches in a network or a lack of data to discriminate between multiple branches that attained significant PINE *P*-values.

Overall, given a variant *x*, the PINE algorithm exhaustively calculates PINE *P*-values for each candidate branch $b \in B$ in an input network. When multiple significantly-perturbed branches exist, it is possible to further test the presence of a single leading branch using the leading *P*-value score.

### Synthetic data

#### Data construction

The analysis builds on a group of 13 synthetic signaling networks with *n* = 2, 3, 4 and 5 stimulations (Supplementary Figure S3), as well as on a synthetic genotyping dataset of 100 DNA variants across 100 individuals (assuming homozygous lines and equal probability for sampling one of the two genotypes). We generated synthetic data ‘collections’, each collection consists of 130 ‘sample datasets’: 10 sample datasets for each of the 13 networks. In particular, for each network only five sample datasets are affected by a variant (*w* > 0) whereas the remaining five samples are not affected by any variant (*w* = 0). Thus, in total, a single collection consists of 65 perturbed and 65 non-perturbed sample datasets. Each of the sample datasets was created as follows: we first randomly chose one DNA variant and one perturbed branch. We then generated the group of genes and positioned them as targets of the transcription factors that are downstream of the selected perturbed branch (choosing the same number of genes for each such downstream transcription factor). The transcriptional response of each gene was sampled for each individual and stimulation according to three possible Gaussian distributions: no-response, $a$-response and $\bar a$-response Gaussians (using $\mu _0$,$\mu _a$,$\mu _{\bar a}$ and $\sigma _0$,$\sigma _a$,$\sigma _{\bar a}$, respectively). The no-response Gaussian indicates the absence of a stimulation signal, whereas the $a$-response and $\bar a$-response Gaussians indicate the response to stimulation in individuals carrying $a$ and $\bar a$ genotypes, respectively. Without loss of generality, we assume that the $a$-carrying individuals exhibit a reduced response compared to the $\bar a$-carrying individuals, thus assuming $\mu _0$ = 0, $\mu _{\bar a}$ = 2 and $\mu _0$< $\mu _a$< $\mu _{\bar a}$. The ‘effect size’ of the variant (the strength of difference between the two genotypes) is therefore defined as *w =* $\mu _{\bar a}$ *-* $\mu _a$. Notice that while $\mu _0$ and $\mu _{\bar a}$ are fixed (0 and 2, respectively), $\mu _a$ is determined according to the desired effect size.

We generated a large number of synthetic collections, each collection was constructed using a particular number of individuals (*m* = 2, 10, 20, 30, 50 or 100), a certain number of genes that associate with a given variant (*t =* 2, 10, 20, 30, 50 or 100) and a selected ‘effect size’ value (*w =* 0.1, 0.2, 0.3, 0.4 or 0.5); unless stated otherwise, we used $\sigma _0 = \sigma _a = \sigma _{\bar a}$ = 0.5, 1, 1.5 or 2.

To further test inaccuracies introduced by altering the information related to genes downstream to the perturbed branches, we generated two additional types of such synthetic samples: in a ‘gene exchange’ sample, a certain percentage of the genes downstream to the perturbed branch are switched with genes that are *not* downstream to that branch; alternatively, in a ‘false targets’ sample, a certain percentage of the genes downstream to the perturbed branch are *not* affected by any variant (*w* = 0).

#### Comparison of methods

We compared the performance of the PINE *P*-value to that of two alternative methods—the InCircuit algorithm (6) and a dummy random solution. We chose to focus on the PINE *P*-value (rather than on the joint usage of the PINE and leading *P*-value scores) based on the observation that the overall performance of PINE can only be improved with additional discriminative scores. The InCircuit algorithm uses two input parameters: a cutoff for a significant association and a cutoff for determining enrichment of genes downstream of a certain transcription factor in a network. We used association cutoff 0.1 and enrichment cutoff 0.9 that performed better than any other parameter combination (Supplementary Figure S4; see ‘Performance analysis’ section below). The random method selects the branch arbitrarily, assuming equal probability for all branches in the given network. Whereas our algorithm calculates a PINE *P*-value for each of the candidate branches, the output of the InCircuit and random methods is a deterministic (binary) decision about each of the branches. Notably, in our multiple-stimulation synthetic dataset, many of the associations exist in one stimulation but not in another stimulation. Several existing methods (4,5) assume a single-condition dataset and hence could not be applied on our synthetic dataset.

#### Performance analysis

The quality of prediction was evaluated using standard performance metrics. Each of the synthetic data collections comprised 130 sample datasets, in which each sample *c* associates with a specific selected perturbed branch *c_b_*. Half of the samples were created using zero effect size (*w* = 0; denoted ‘non-perturbed’ branch *c_b_*), whereas the remaining 65 samples were generated using a non^_-_^zero effect size (*w* > 0; denoted ‘perturbed’ branch *c_b_*). Given a sample *c*, each of the compared algorithms was applied on branch *c_b_*, providing either a ‘positive’ or a ‘negative’ decision on this branch (for the PINE algorithm, we used a PINE *P*-value cutoff of 0.05 to split between positives and negatives). The true-positive (TP), false-positive (FP), true-negative (TN) and false-negative (FN) measures were defined according to the correct (perturbed or non-perturbed) simulated solution compared to the positive and negative predictions, allowing to calculate the ‘accuracy score’ as (TP + TN)/(TP + TN + FP + FN). Notably, using the PINE *P*-value we can also calculate the area under the receiver operating characteristic curve (the ‘AUC score’).

### Real data

We compiled a previously published bone marrow-derived DCs dataset (6) of gene expression measured at steady state and at 6 h after *in vitro* pathogenic stimulations with PAM, LPS and poly I:C (agonists of TLR2, TLR4 and TLR3 or RIG-I receptors, respectively), spanning 43 BXD mouse strains. The dataset was measured using a meso-scale assay (the NanoString nCounter system; (15)) and consists of only 422 genes that were previously selected so as to represent associations with a large variety of genetic variants regard­less of their effect sizes (6). The response of a gene (in a given strain and following a specific stimulation) was calculated as the log-ratio between the expression level after exposure to stimulation and at steady state. The input signaling network includes the TLR and RIG-I signaling pathways (9,10) and consists of two transcription factors (NFκB and Irf3), as shown in Supplementary Figure S5A. The formal Bayesian network model is presented in Supplementary Figure S5B (network confidence level *β* = 0.995). To allow biological interpretability, the results are presented using signaling pathways as visualized in Figure 5; the visualization includes seven different signaling pathways, each of these pathways reflects one or a few Bayesian network branches that share a certain proxy node, as shown in Supplementary Figure S5C. We conducted separate analyses for each of five DNA variants #1−#5 (Supplementary Table S1), where the variants, the associated genes and their network positions downstream to NFkB and Irf3 were previously identified in (6). Genotyping data were downloaded from WebQTL (8). The analysis was performed using 2000 permutations for all *P*-values calculations. Unless stated otherwise, the reported leading *P*-value of a branch is the largest (least significant) leading *P*-value of that branch (across comparisons with all remaining branches); all reported *P*-values were corrected for multiple testing using the Holm-Bonferroni method (16).

**Figure 3.**
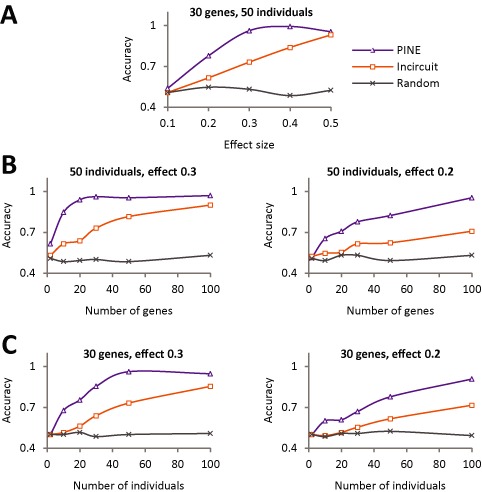
Performance analysis on synthetic data. (**A**) Shown is the accuracy metric (*y*-axis) across different effect sizes (*x*-axis), using synthetic datasets of 30 genes, 50 individuals and standard deviation 1. (**B**,**C**) The accuracy scores for varying numbers of genes (**B**; using 50 individuals) and individuals (**C**; using 30 genes) for synthetic datasets with effect size of 0.3 (left) and 0.2 (right) using standard deviation 1. In all cases, results are shown for three methods: the PINE, InCircuit and random selection algorithms (color coded; using PINE *P*-value cutoff = 0.05). The plots indicate the superiority of the PINE score over the InCircuit and random methods, showing PINE's robustness to varying data parameters.

**Figure 4.**
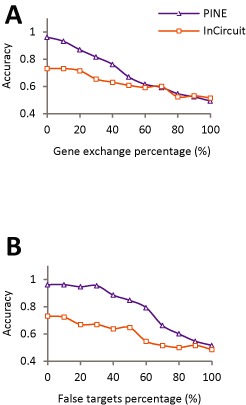
Robustness to erroneous prior knowledge. Testing erroneous prior knowledge due to switching between genes downstream to the perturbed branch and genes that are not downstream to that branch in the network (‘gene exchanges’, **A**) or due to introducing non-perturbed genes downstream to the perturbed network branches (‘false targets’, **B**). Shown is the accuracy metric (*y*-axis) across synthetic data collections carrying varying percentages of gene exchanges (**A**) or false targets (*x*-axis, **B**) for the PINE (purple) and InCircuit (orange) methods, assuming 30 genes, 50 individuals, effect size 0.3 and standard deviation 1 (using PINE *P*-value cutoff = 0.05). The plots indicate the robustness of PINE compared to the InCircuit method across different percentages of gene exchanges or false gene targets.

**Figure 5.**
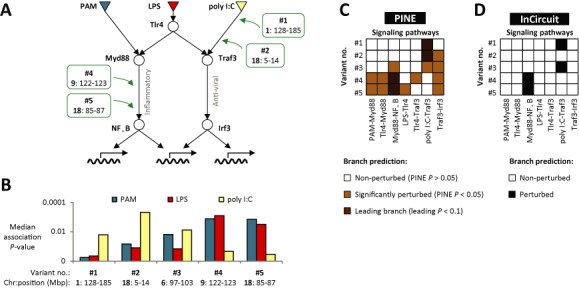
Variation in the TLR/RLR signaling network of mouse DCs in response to pathogenic components. (**A**) The TLR/RLR signaling pathways, together with the leading perturbed branches inferred by PINE framework for variants #1, #2, #4 and #5 (as detailed in **C**). The Bayesian network formalism is shown in detail in Supplementary Figure S5. (**B**) Stimulation-dependent associations of gene targets and genetic variants. The histogram shows the median genetic association *P*-value (*y*-axis, ANOVA test, log-scaled) over all genes that are associated with a DNA variant (*x*-axis) following at least one stimulation. *P*-values were calculated using transcription data following the PAM (blue), LPS (red) and poly I:C (yellow) stimulations. Variants #1-#3 have the strongest associations with their targets following poly I:C stimulus, whereas associations of variants #4-#5 are mostly pronounced following the PAM and LPS stimulations. (**C**) A perturbed branch matrix based on the PINE algorithm. The matrix consists of a color-coded representation of PINE's prediction for a particular branch (column) and a variant (row): white: non-perturbed branches (PINE *P* > 0.05); light brown: significantly-perturbed branches (PINE *P* < 0.05); dark brown: significant leading branches (leading *P* < 0.05 for variants #1 and #2) or leading *P* < 0.06 (for variants #4 and #5). (**D**) A perturbed branch matrix based on InCircuit's binary predictions, where black/white indicates predicted perturbed/non-perturbed branch.

## RESULTS

### PINE accurately identifies perturbed branches in signaling networks

We used synthetic datasets to evaluate the performance of the PINE algorithm. The synthetic data is based on 13 different signaling networks that are triggered by 2–5 extracellular stimulations (Supplementary Figure S3). For the generation of synthetic gene expression data, we used four parameters: (i) number of individuals (2, 10, 20, 30, 50 and 100); (ii) number of genes (2, 10, 20, 30, 50 and 100); (iii) genetic effect size (0.1, 0.2, 0.3, 0.4 and 0.5); and (iv) standard deviation (0.5, 1, 1.5 and 2). In all cases, different genetic influences were generated under different stimulations, depending on the selected position of the variant and the positions of its target genes within the synthetic signaling network (Materials and Methods section).

We first tested the PINE *P*-value score across varying method parameters using the AUC score (Materials and Methods section). The analysis was performed using two data collections that have either effect size of 0.2 or 0.3 (30 genes, 50 individuals and standard deviation = 1 in all collections), suggesting that 150 permutations are sufficient for attaining valid PINE *P-*values (Supplementary Figure S6A, e.g. Supplementary Figure S7). AUC levels improve with increasing confidence in the signaling network (Supplementary Figure S6B), consistently with the design of the PINE framework based on prior knowledge about the biological system under study. In the following we therefore first analyze performance in the case of high confidence in the input network (*β* = 0.995) and then present the performance in cases of inaccuracies in this input (in all cases we use 150 permutations).

We compared the PINE *P*-value score with two alternative approaches: the InCircuit algorithm and a random selection method (Materials and Methods section). Hereafter, the quality of predicted perturbed branches was evaluated using an accuracy score, defined as the ratio between true predictions (true positives and true negatives) and the total number of predictions; the higher the accuracy, the better the performance (assuming PINE *P*-value cutoff = 0.05; ‘Materials and Methods’ section). The InCircuit algorithm was applied using a combination of parameters that attained the best performance (Materials and Methods section). We first demonstrate the results using synthetic data of 50 individuals and 30 genes across varying effect size values, with standard deviation of 1 (Figure 3A). Both PINE and InCircuit appear to best perform with high effect sizes, but the InCircuit and random solutions attain comparably lower accuracy scores in all effect sizes. PINE was comparable to the InCircuit method only in the case of high effect size as 0.5, where there is a clear difference between the genotypes. Similar results were obtained when using varying numbers of genes and individuals (Figure 3B and C) and using different standard deviation values (Supplementary Figure S8): although PINE's accuracy is reduced with a lower number of genes or individuals and with a higher standard deviation, it still maintains higher accuracy than those of the InCircuit (and random) algorithm.

The simulations further allow us to assess the robustness of the PINE scoring scheme to erroneous prior information related to genes downstream to the perturbed branches. To this end, we generated synthetic ‘gene exchange’ collections with varying percentages of genes downstream to the perturbed branch that are switched with genes that are not downstream to that branch (0–100%, ‘Materials and Methods’ section). The accuracy values of both PINE and InCircuit declined with higher percentages of exchanged genes; notably, however, PINE maintained higher accuracy than InCircuit, comparable only at a high percentage (>60%) of exchanged genes (Figure 4A). For example, PINE's and InCircuit's accuracy drops below 0.8 and 0.65 when more than 40% of the genes were exchanged. Next, we further tested synthetic ‘false targets’ collections with varying percentages of genes that are located downstream to the perturbed branch but are not affected by the genetic variant (0–100% of the target genes, ‘Materials and Methods’ section). Notably, PINE revealed a stronger robustness for high percentage of false targets compared to the InCircuit method (Figure 4B). For example, for 40% false target genes, accuracy = 0.88 and 0.64 for the PINE and InCircuit methods, respectively.

Taken together, our results suggest that the PINE *P*-value is a robust score, performing well over a broad range of effect sizes, numbers of genes, numbers of individuals and standard deviations.

### Identification of perturbed branches in murine DCs in response to pathogenic components

We next turned to investigating the mechanisms of variants in the TLR/RLR signaling pathway in DCs. Our network model comprised of TLRs and RLRs that recognize pathogenic ligands and trigger activation of the inflammatory and antiviral pathways as well as transcription regulators such as NFκB and Irf3 (Figure 5A and Supplementary Figure S5A; ‘Materials and Methods’ section). Within this model we used PINE to test a list of seven candidate network branches (Supplementary Figures S5B and C). We analyze the transcription responses of 422 genes in bone marrow-derived DCs after stimulations with three pathogenic-like ligands (PAM, LPS and poly I:C), spanning 43 genotyped recombinant inbred mouse strains (data from (6), ‘Materials and Methods’ section). We demonstrate the application of PINE in the cases of five known DNA variants (termed #1−#5) and their stimulation-dependent associated genes (91 genes, 22% of 422; Supplementary Table S1 and Figure 5B). Notably, PINE provides significant predictions of perturbed branches for each of these variants (PINE *P* < 0.05), with significant leading branches for two variants (#1 and #2, leading *P* < 0.034) and a leading tendency (though not significant) for branches of two other variants (leading *P* < 0.052 [#4] and *P* < 0.06 [#5]; Figure 5C and Supplementary Table S1). For each variant we first present PINE's predictions, and then use it to exemplify the PINE methodology and its ability to reconstruct biologically-relevant hypotheses.

Variant #1 exemplifies the method's ability to identify a single network branch as a significantly-perturbed branch that likely propagates the signal to its 30 downstream genes. We first augmented the signaling network with 30 genes that are associated with variant #1. The genes were added downstream of the transcription factors Irf3, NFκB or both, based on the response of each of the genes to the various stimulations (Supplementary Table S1). For example, 16 genes (e.g. *Iigp2*) were positioned downstream of Irf3 based on their transcriptional response to LPS and poly I:C stimuli but not to PAM. Similarly, 11 genes (e.g. *Stat2*) were positioned downstream of both Irf3 and NFκB according to their significant response to all three triggering stimulations. By applying PINE on this expanded network, only a single branch—the poly I:C–Traf3 branch—attained a significant PINE *P*-value (PINE *P* < 0.015). In agreement, the poly I:C–Traf3 branch achieved significant leading *P*-values compared to any other branch (leading *P* < 0.034; Figure 5C and Supplementary Table S2). This prediction for variant #1 is supported by a previous study that identified the likely causal gene *Rgs16* and demonstrated its specific functionality following poly I:C but not PAM or LPS stimuli (6), which is indeed the stimulation in the upstream region of the poly I:C–Traf3 branch.

Variant #2 demonstrates the ability of PINE to pinpoint a single leading branch among several significantly-perturbed branches. The poly I:C-Traf3 is suggested as the leading branch (PINE *P* < 0.015, leading *P* < 0.034, Figure 5C), further supported by the match between the poly I:C stimulation, following which strong associations of the target genes to variant #2 are found (Figure 5B) and the upstream stimulations signature of the poly I:C-Traf3 branch (Figure 5A). One additional branch (Traf3-Irf3) that responds to both poly I:C and LPS is suggested as a non-leading significantly-perturbed branch (PINE *P* < 0.027, leading *P* > 0.1; Supplementary Table S2), consistently with the partial agreement between the upstream stimuli of this branch (poly I:C and LPS) and the poly I:C-dependent associations of target genes to this variant (Figure 5A and B). All remaining branches, which respond to PAM or LPS (but not to poly I:C), did not achieve significant *P*-values (PINE *P* > 0.05), as expected (Figure 5C).

Variant #3 illustrates PINE's ability to identify several significantly-perturbed branches (poly I:C-traf3, Traf3-irf3, Myd88-NFκB, PINE *P* < 0.03 in all cases) whereas none of these branches is a leading branch (leading *P* > 0.1; Figure 5C and Supplementary Table S2). Indeed, the target genes of variant #3 are mostly associated with both PAM and poly I:C (Figure 5B), which do not appear together in the upstream region of any candidate branch; instead, each of these stimuli appears separately in the upstream region of the predicted branches, supporting PINE's identification of several partly significant branches rather than a single leading branch.

Variants #4 and #5 indicate that our methodology can identify downstream network branches that integrate information from several stimuli. The suggested leading branch (Myd88-NFκB, leading *P* < 0.052 [#4], 0.06 [#5]) is affected by both PAM and LPS (Figure 5A, C and Supplementary Table S2) and the target genes are also mostly associated with variants #4-#5 following the same combination of stimuli (Figure 5B), thus supporting the placement of these variants in the Myd88-NFκB branch.

Overall, the results demonstrate the utility of using both the PINE and leading *P*-values: the leading *P*-value is tailored for identifying a single leading branch (using the PINE *P*-value scores as a filtering stage; variants #1,#2,#4,#5), whereas the PINE *P*-value is unique in providing insights about the presence of two or more plausible perturbed branches (variant #3).

We next compared the PINE and the InCircuit algorithms (6) (Figure 5C versus D). Of the four branches that were identified by InCircuit in variants #1−#5, PINE provided support for only three branches (leading *P* < 0.034 [#1], 0.052 [#4], 0.06 [#5]). Importantly, whereas InCircuit predicted a single branch for variant #3, PINE suggested that three alternative significantly-perturbed branches exist but none of them is substantially better than the remaining two branches (PINE *P* < 0.03; leading *P* > 0.1). Variant #3 therefore exemplifies PINE's ability to compare between competing branches in a quantitative manner, unlike the (qualitative) InCircuit approach. Furthermore, PINE succeeded in finding additional significant leading branch (for variant #2; leading *P* < 0.034) that could not be detected by the InCircuit algorithm. These results exemplify the advantage of PINE over extant methods in that it enables users to systematically and reliably evaluate statistical significance values for candidate branch hypotheses.

## DISCUSSION

Despite the importance of DNA variants in mammalian genomes, an in-depth understanding of their effects on signaling pathways has remained elusive. In fact, the computational tools required to predict which network branches are perturbed by such variants are still in their infancy. In a recent study it was proposed that this problem can be tackled by utilizing genetic genomic information across multiple stimulations (6). That approach yielded testable hypotheses about the effects of variants on a molecular network, but could not evaluate their statistical meanings.

In this study we introduce PINE, a novel computational approach for the statistical evaluation of branches through which a DNA variant perturbs a given signaling network. Significant hypotheses can then be selected for followup experiments. We first applied PINE on synthetic data across multiple stimulations, exemplifying its utility for different data parameters, where we found PINE predictions to be highly accurate and performing better than existing methods (Figure 3). We further showed that PINE is robust to inaccuracies in the prior knowledge about the target genes downstream to the perturbed branches, unlike previous approaches (Figure 4). Finally, we examined the utility of PINE on a mouse dataset, consisting of five DNA variants that underlie transcriptional response of immune DCs during three pathogenic stimulations. The analysis supports three previously identified perturbed branches (for variants #1, #4, #5), predicted a new statistically significant perturbation in a branch (for variant #2) and concluded that it is impossible to distinguish the best-fit branch for one additional variant (#3, Figure 5A and C).

Our work offers the basis for future studies exploring the molecular mechanisms underlying complex traits. First, our framework can be further developed to reveal the position of gene–gene interactions within the signaling network. Secondly, PINE relies on prior knowledge about the signaling pathways that are downstream to the environmental stimulations under study. Several different databases contain manually-curated signaling networks (e.g. KEGG (17), Reactome (18) and the Ingenuity Knowledge Base (19)) that can be used as prior knowledge. Although inaccuracies in the signaling networks led to reduced PINE performance, the accuracy loss was much lower compared to those of existing methods (Figure 4). In the future, we hope that the PINE approach will lead to development of more sophisticated algorithms with higher robustness to errors in prior knowledge. For example, it may be possible to revise inaccuracies in the signaling network based on inter-individual variation in transcriptional responses.

Thirdly, application of PINE on different datasets and their corresponding networks will prove that the method is generic and compatible with different biological processes. We are particularly interested in extending our approach to handle outbred strains and human responses, which present challenges such as heterozygosity and population structure. PINE is based on measurements of transcriptional responses across multiple stimulations, thus requiring RNA profiling not only in the presence of various stimulations but also in the absence of stimulation. As more appropriate datasets become available, applying the PINE method may yield new insights about the specific molecular mechanisms through which genetic variants perturb signaling networks.

## SUPPLEMENTARY DATA

Supplementary Data are available at NAR Online.

SUPPLEMENTARY DATA
