# Supplementary material for: A statistical framework for revealing signaling pathways perturbed by DNA variants
Source: Nucleic Acids Res. 2015 Mar 12;43(11):e74. doi: 10.1093/nar/gkv203 (PMC4477643; doi:10.1093/nar/gkv203)
Supplement: SUPPLEMENTARY DATA [file supp_43_11_e74__index.html]

A statistical framework for revealing signaling pathways perturbed by DNA variants — A statistical framework for revealing signaling pathways perturbed by DNA variants — SUPPLEMENTARY DATA 

# A statistical framework for revealing signaling pathways perturbed by DNA variants

## SUPPLEMENTARY DATA

**Files in this Data Supplement:**

- SUPPLEMENTARY DATA
