# Supplementary material for: A statistical framework for revealing signaling pathways perturbed by DNA variants
Source: Nucleic Acids Res. 2015 Mar 12;43(11):e74. doi: 10.1093/nar/gkv203 (PMC4477643; doi:10.1093/nar/gkv203)
Supplement: SUPPLEMENTARY DATA [file supp_gkv203_nar-03148-met-n-2014-File007.pdf]

A statistical framework for revealing signaling pathways perturbed by DNA variants

Roni Wilentzik and Irit Gat-Viks*

Department of Cell Research and Immunology, The George S. Wise Faculty of Life Sciences, Tel Aviv University, 6997801 Tel Aviv, Israel

* Corresponding author: iritgv@post.tau.ac.il (IGV)

Supplementary Figures

- Supplementary Figures 1-8 Legends (p. 2-4)
- Supplementary Figures 1-8 (p. 5-12)

Supplementary Tables

- Supplementary Table 1 (p.13)
- Supplementary Table 2 (p.14)

Supplementary Figures Legends

Supplementary Figure 1: DNA variant perturbing a signaling pathway. Top: Presented is a signaling network triggered by two environmental stimulations (s_1 and s_2, triangles) whose signals propagate through signaling components (circles) towards the regulated genes (g_1 and g_2, squares) along various network branches (b_1-b_5, edges). The DNA variant *x* (green) perturbs branch b_2, which is upstream of g_1 and g_2 and downstream of s_2 but not downstream of s_1. **Bottom**: Presented are the predicted response values for each of the network components (high/low predicted response – dark/light orange) assuming a certain stimulation and allele (indicated as splits in the hierarchical tree). The illustration shows that variant *x* affects the response of g_1 and g_2 but only following s_2 stimulation. The perturbation following stimulation s_2 can be seen on the two rightmost panels, where genes g_1 and g_2 have low response (light orange) under the '*A*' genotype but high response (dark orange) under the '*G*' genotype (since we focus on fully homozygous lines, the allele in one chromosome is sufficient).

Supplementary Figure 2: Schematic illustration of the MetaReg framework (12,13) for Bayesian network modeling of a biological system. The Bayesian network model of a signaling network consists of molecular variables and the relationships between them (left panel) along with a conditional probability function for each of the variables (right panel). **Left**: A model consists of variables (nodes), representing molecular entities, and their regulatory relations (edges). Here, the variables are discrete and can be assigned one of two states – either 0 or 1. **Middle**: Each variable is associated with a deterministic logical function that is formalized on the basis of quantitative prior knowledge and describes the logic of regulation for this variable. For example, according to the induction logic, the state of variable *z* is determined by the maximum among the states of its regulators *x* and *y*, that is $z=max(x,y)$ (bottom); the state of *w* equals the state of its regulator *y*, since it has only one regulator (top). **Right**: The deterministic logics are further translated into conditional probabilities using a fixed 'network confidence level' parameter β (here, β=0.995), which reflects our level of certainty in the deterministic outcome. The diagram is modified from Ref. 12.

Supplementary Figure 3: A collection of 13 synthetic signaling networks. The collection consists of all networks that contain: (i) two, three, four or five stimulations; (ii) two transcription factors that differ in their combination of upstream stimulations; and (iii) the input degree of all variables is either 0, 1 or 2. Illustration is as in **Supplementary Fig. 1**.

Supplementary Figure 4: Parameter selection for InCircuit. Shown are the accuracy scores (*y*-axis) across different enrichment cutoff parameters (*x*-axis) and four alternative association cutoff parameters (color coded), calculated for synthetic collections of 30 genes, 50 individuals, effect size 0.3 and standard deviation 1 when using the original collections (**A**), 50% of gene exchange (**B**) and 50% of false targets (**C**). The best combination of parameters (association cutoff = 0.1 and enrichment cutoff = 0.9 in all cases) was used in InCircuit's analyses.

Supplementary Figure 5: Construction of the TLR/RLR signaling network. (**A**) **Prior knowledge about the TLR/RLR signaling pathways.** Shown is a network illustration of the TLR/RLR signaling pathways, constructed on the basis of well-established prior knowledge (9,10). The network is triggered by three stimulations: the bacterial components PAM and LPS, which trigger the Tlr2 and Tlr4 receptors respectively, and the viral-like poly I:C component, which triggers the Tlr3 and the RIG-I/Mda5 receptors. The response of the downstream genes is regulated by the NFκB and Irf3 transcription factors. (**B**) **The Bayesian network model.** The Bayesian network was constructed based on the pathways from **A** as follows: First, the network from **A** was simplified by merging non-informative chains of branches (top panel). For example, the six branches from Myd88 to NFκB in **A** were merged into a single Myd88-NFκB branch in **B**. Second, mock nodes were added to allow examination of all branches (gray circles; **Methods**). Finally, a local probability distribution was added to each of the variables (bottom panel; as detailed in **Supplementary Figure 2** and assuming β=0.995). (**C**) **Signaling pathways and their proxy nodes**. The table provides details about each of signaling pathways that appear in **Figure 5A** (column 1). For each signaling pathway, shown are the particular branch identifiers and proxy nodes in the formalized Bayesian network model from **B** (columns 2 and 3, respectively).

Supplementary Figure 6: The performance of the PINE *P*-value across method parameters. Shown is the AUC metric (*y*-axis) across varying number of permutations (**A**) and network confidence levels β (**B**, *x*-axis). Results are shown for two different effect sizes (color coded) using 30 genes, 50 individuals and standard deviation 1.

Supplementary Figure 7: Characterization of the PINE *P*-value score. (**A**) Shown is the rate of false discoveries (FDR = FP/(TP+FP); *y*-axis) across varying bins of *P*-value cutoff (*x*-axis). (**B**) Shown is the 'fraction of true positives' (black) and 'fraction of false positives' (gray; *y*-axis), estimated as the percent of true or false positives out of the tests that attained a *P*-value within the certain range of the bin (*x*-axis). The two plots are shown for a synthetic collection with 30 genes and 50 individuals, effect size 0.3 and standard deviation 1. The plots indicate that the lower the *P*-value range, the lower the FDR and the fraction of false positives, and the higher the fraction of true positives.

Supplementary Figure 8: Performance analysis across varying levels of noisy measurements. The accuracy values for a synthetic dataset of 30 genes and 50 individuals. (**A**) Shown is the accuracy metric (*y*-axis) across different standard deviation values ($\sigma = \sigma_0 = \sigma_a = \sigma_{\bar{a}}$; *x*-axis) for effect sizes 0.1 (left), 0.3 (middle) and 0.5 (right). (**B**) Shown is the accuracy metric (*y*-axis) across different effect sizes (*x*-axis) for different combinations of standard deviation values for the *'no-response'*, 'a *-response*' and '$\bar{a}$ *-response*' Gaussians (see **Methods**): $\sigma_0 = 1, \sigma_a = \sigma_{\bar{a}} = 0.5$ (left), $\sigma_0 = 1.5, \sigma_a = \sigma_{\bar{a}} = 1$ (middle) and $\sigma_0 = 2, \sigma_a = \sigma_{\bar{a}} = 1.5$ (right). In both **A** and **B**, results are shown for both PINE and InCircuit (color coded), suggesting better performance of PINE across varying standard deviation values.

Supplementary Figure 1

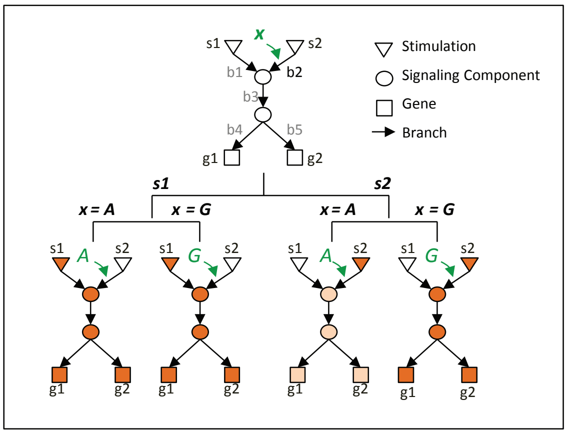

Supplementary Figure 2

Prior knowledge

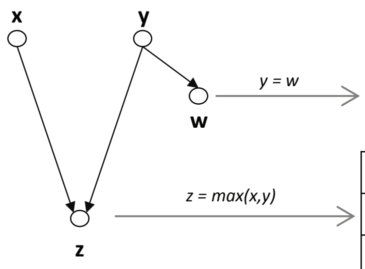

Discrete formalization of logics

y	w (y)
0	0
1	1

x	y	z(y,x)
0	0	0
0	1	1
1	0	1
1	1	1

Network confidence level β = 0.995

Probabilistic formalization of logics

y	w=0	w=1
0	0.995	0.005
1	0.005	0.995

x	y	z=0	z=1
0	0	0.995	0.005
0	1	0.005	0.995
1	0	0.005	0.995
1	1	0.005	0.995

Supplementary Figure 3

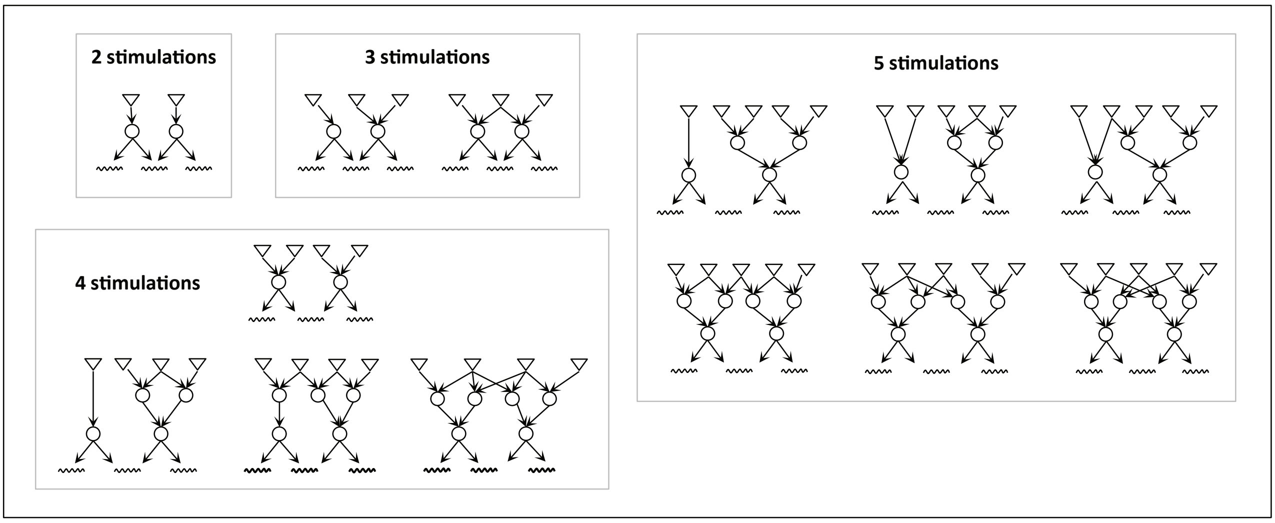

Supplementary Figure 4

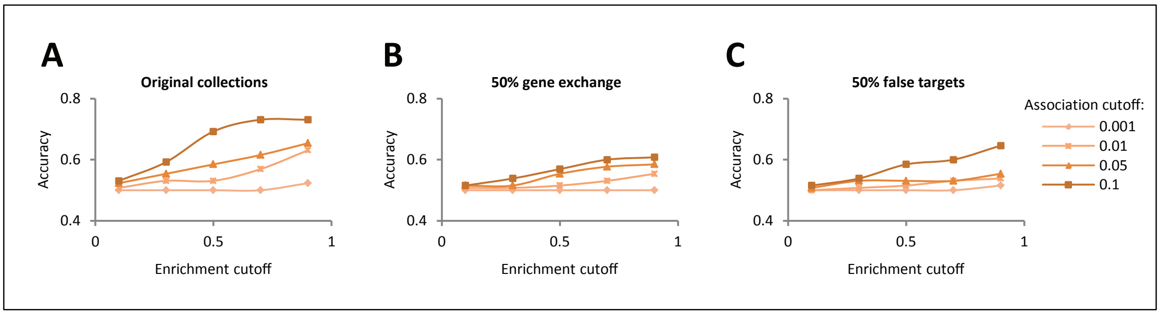

Supplementary Figure 5

A

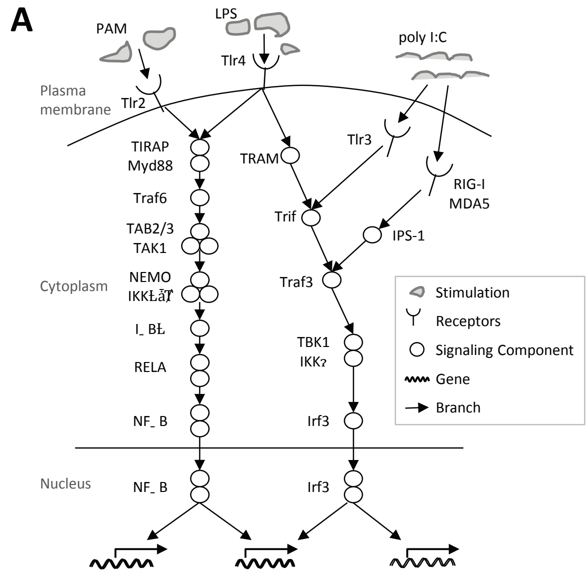

B

Signaling component	Regulation logic
Tlr2, Tlr3/RIG-I/MDA5, Tlr4, NF_ B, Irf3, Mock1, Mock2	*y = w*
Myd88, Traf3	*z = max(x,y)*

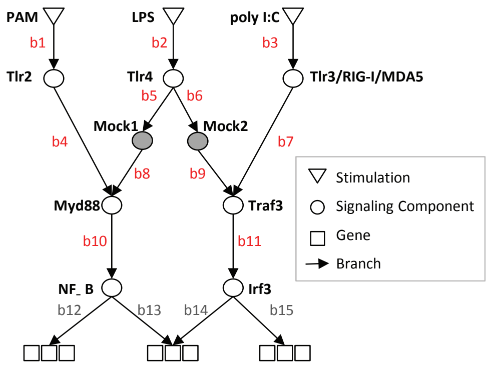

C

Signaling pathway	Branch identifier	Proxy node
PAM – Myd88	b1 + b4	Tlr2
Myd88 – NF_ B	b10	Myd88, NF_ B
LPS – Tlr4	b2	Tlr4
Tlr4 – Myd88	b5 + b8	Mock1
Tlr4 – Traf3	b6 + b9	Mock2
poly I:C – Traf3	b3 + b7	Tlr3/RIG-I/MDA5
Traf3 – Irf3	b11	Traf3, Irf3

Supplementary Figure 6

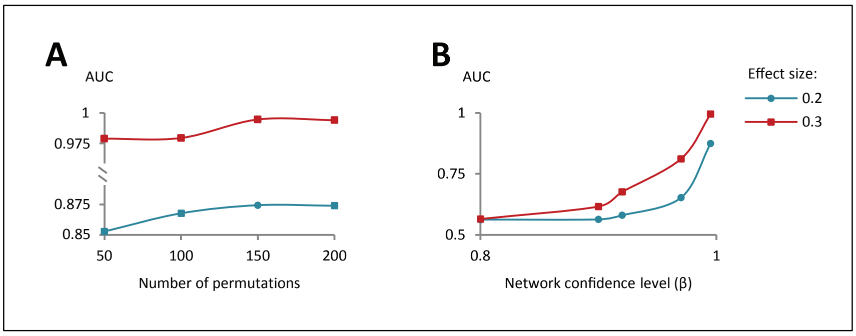

Supplementary Figure 7

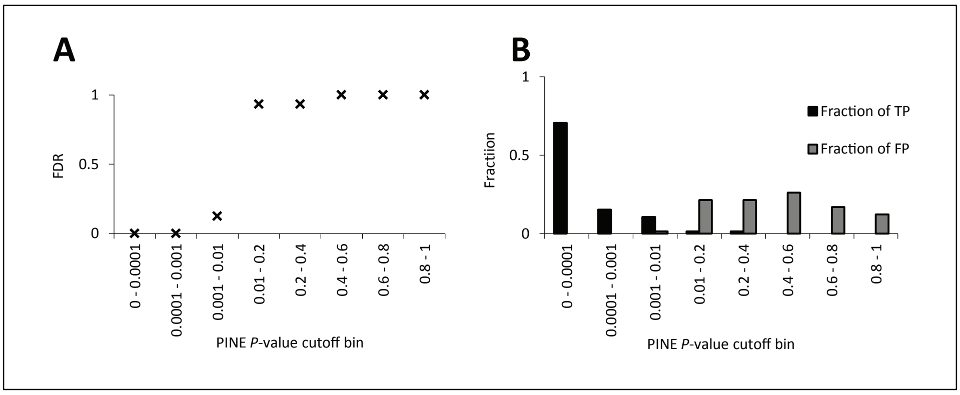

Supplementary Figure 8

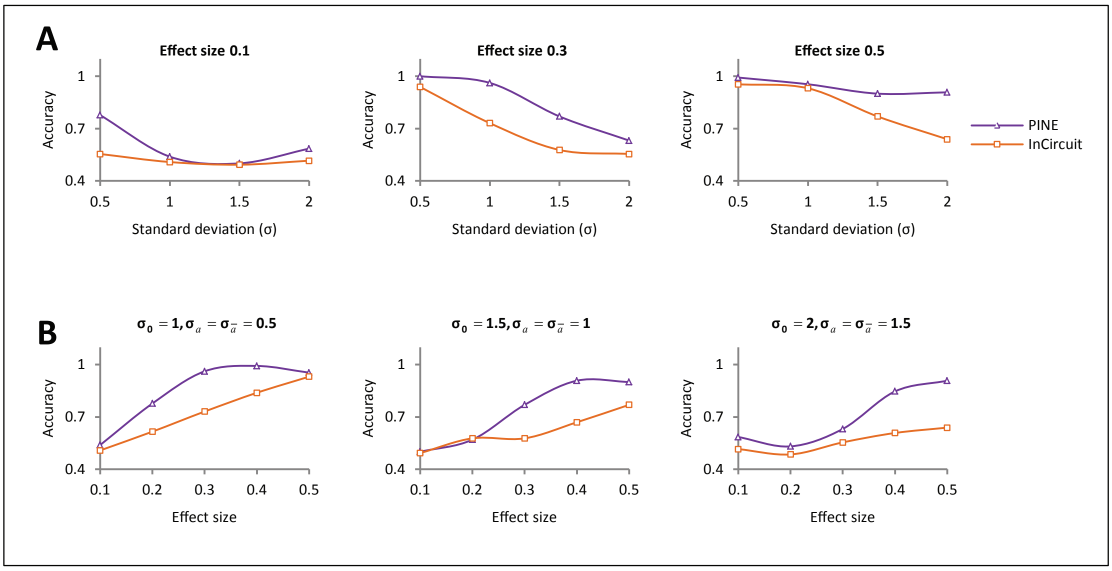

Supplementary Tables

Supplementary Table 1: DNA variants acting on transcriptional responses of multiple genes in murine dendritic cells. For each known DNA variant (1st column; based on Ref. 6.), the table presents the genomic position of the variant (2nd column), genes that associate significantly with this variant (3rd column; from Ref. 6) along with their network positions (from Ref. 6: *NFκB regulation; ^NFκB and Irf3 regulation; +Irf3 regulation), and the leading perturbed signaling pathways predicted by PINE algorithm together with their (maximal) leading *P*-values (4th column, all reported *P*-values are Holm-Bonferroni corrected).

Variant no.	Genomic position (Mbp)	Gene symbol	PINE's leading branch prediction
#1	**1**:128-185	*Clec5a**, *Hdac5**, *Rusc2**, *Stat2*^, *Ints4*^, *Csf1*^, *Timeless*^, *Rbm43*^, *Tgif1*^, *Myd88*^, *Tlr2*^, *Smpdl3b*^, *Vwf*^, *Tnf*^, *Iigp2*+, *Slfn8*+, *Ifi44*+, *Ddx60*+, *Ifit2*+, *Ifit3*+, *Irf7*+, *Dhx58*+, *Cox18*+, *Nmi*+, *Sp100*+, *Daxx*+, *Iigp1*+, *Arid5a*+, *Irf2*+, *Trim21*+	poly I:C-Traf3 (leading *P* < 0.034)
#2	**18**:5-14	*Socs2**, *Med21**, *Cd1d1**, *Ypel3**, *Cd1d2**, *Hmgn3*^, *Idi1*^, *Plscr1*^, *Ifnb1*^, *Pmvk*^, *Il12rb2*^, *Ptger4*^, *Dusp16*^, *Clcn7*^, *Kctd14*^, *Gpr109a*^, *Ifna2*+, *BC013712*+, *Ifna4*+, *Cxcl9*+	poly I:C-Traf3 (leading *P* < 0.034)
#3	**6**:97-103	*Zmat3**, *Prmt3*^, *Pdk1*^, *Ikbke*^, *Slc6a4*^, *Exosc5*^, *Tlr7*+, *Trim25*+, *Atad3a*+, *Ddx18*+, *Ftsj3*+, *Bysl*+, *Irf8*+	Nonsignificant
#4	**9**:122-123	*Nlrp3**, *Ehd1**, *Cxcr4**, *Gadd45a**, *Spred1**, *Dhrs3**, *Six1*^, *Eps8*^, *Rasgrp1*^, *Nfkb2*^, *Tsc22d3*^, *Lfng*^, *Tef*^, *Socs6*^, *E2f5*^, *Gtf2e2*^, *Fam107b*^, *Net1*+	Myd88-NFκB (leading *P* < 0.052)
#5	**18**:85-87	*Areg**, *Ereg**, *Ripk3**, *Ptgs1**, *Nfe2**, *Batf**, *Rnh1*^, *Ugcg*^, *Trem1*^, *Spry1*^, *C5ar1*^, *Il15ra*+	Myd88-NFκB (leading *P* < 0.06)

Supplementary Table 2: Leading branches. Shown is a matrix where each cell represents the leading *P*-values for a given branch b' perturbed by a certain variant (rows) against an alternative candidate branch b'' perturbed by the same variant (columns) (see equation (5) in **Methods** for details). In this table, leading branches are those branches whose maximal leading *P*-value < 0.1 across an entire row (marked in bold).

Variant no.	Network branch	PAM-Myd88	TLR4-Myd88	Myd88-NFkB	LPS-TLR4	TLR4-TRAF3	PolyIC-TRAF3	TRAF3-IRF3
#1	PAM-Myd88		>0.1	>0.1	>0.1	>0.1	>0.1	>0.1
#1	TLR4-Myd88	>0.1		>0.1	>0.1	>0.1	>0.1	>0.1
#1	Myd88-NFkB	>0.1	>0.1		>0.1	>0.1	>0.1	>0.1
#1	LPS-TLR4	>0.1	>0.1	>0.1		>0.1	>0.1	>0.1
#1	TLR4-TRAF3	>0.1	>0.1	>0.1	>0.1		>0.1	>0.1
#1	**PolyIC-TRAF3**	**0.034**	**0.034**	**0.034**	**0.034**	**0.034**		**0.034**
#1	TRAF3-IRF3	>0.1	>0.1	>0.1	>0.1	>0.1	>0.1	
#2	PAM-Myd88		>0.1	>0.1	>0.1	>0.1	>0.1	>0.1
#2	TLR4-Myd88	>0.1		>0.1	>0.1	>0.1	>0.1	>0.1
#2	Myd88-NFkB	>0.1	>0.1		>0.1	>0.1	>0.1	>0.1
#2	LPS-TLR4	>0.1	>0.1	>0.1		>0.1	>0.1	>0.1
#2	TLR4-TRAF3	>0.1	>0.1	>0.1	>0.1		>0.1	>0.1
#2	**PolyIC-TRAF3**	**0.034**	**0.034**	**0.034**	**0.034**	**0.034**		**0.034**
#2	TRAF3-IRF3	>0.1	0.034	>0.1	0.034	0.034	>0.1	
#3	PAM-Myd88		>0.1	>0.1	>0.1	>0.1	>0.1	>0.1
#3	TLR4-Myd88	>0.1		>0.1	>0.1	>0.1	>0.1	>0.1
#3	Myd88-NFkB	>0.1	>0.1		>0.1	>0.1	>0.1	>0.1
#3	LPS-TLR4	>0.1	>0.1	>0.1		>0.1	>0.1	>0.1
#3	TLR4-TRAF3	>0.1	>0.1	>0.1	>0.1		>0.1	>0.1
#3	PolyIC-TRAF3	0.034	0.034	0.077	>0.1	>0.1		0.034
#3	TRAF3-IRF3	>0.1	>0.1	>0.1	>0.1	>0.1	>0.1	
#4	PAM-Myd88		>0.1	>0.1	>0.1	>0.1	>0.1	>0.1
#4	TLR4-Myd88	0.028		>0.1	>0.1	0.028	0.028	0.028
#4	**Myd88-NFkB**	**0.028**	**0.052**		**0.028**	**0.028**	**0.028**	**0.028**
#4	LPS-TLR4	0.028	>0.1	>0.1		0.028	0.028	0.028
#4	TLR4-TRAF3	>0.1	>0.1	>0.1	>0.1		0.028	0.028
#4	PolyIC-TRAF3	>0.1	>0.1	>0.1	>0.1	>0.1		>0.1
#4	TRAF3-IRF3	>0.1	>0.1	>0.1	>0.1	>0.1	0.052	
#5	PAM-Myd88		>0.1	>0.1	>0.1	>0.1	0.032	>0.1
#5	TLR4-Myd88	>0.1		>0.1	>0.1	0.032	0.032	0.032
#5	**Myd88-NFkB**	**0.06**	**0.032**		**0.06**	**0.032**	**0.032**	**0.032**
#5	LPS-TLR4	>0.1	>0.1	>0.1		0.032	0.032	0.032
#5	TLR4-TRAF3	>0.1	>0.1	>0.1	>0.1		>0.1	>0.1
#5	PolyIC-TRAF3	>0.1	>0.1	>0.1	>0.1	>0.1		>0.1
#5	TRAF3-IRF3	>0.1	>0.1	>0.1	>0.1	>0.1	>0.1	
